# Medical students’ experiences of working with simulated patients in challenging communication training

**DOI:** 10.1186/s41077-022-00230-3

**Published:** 2022-10-10

**Authors:** Johan Isaksson, Julia Krabbe, Mia Ramklint

**Affiliations:** 1 Child and Adolescent Psychiatry and Psychiatry Unit, Department of Medical Sciences, Uppsala University, Uppsala, Sweden; 2 Department of Women’s and Children’s Health, Center of Neurodevelopmental Disorders (KIND), Centre for Psychiatry Research, Karolinska Institutet, Stockholm, Sweden

**Keywords:** Communication skills training, Education, Medical students, Simulated patients, Standardized patients

## Abstract

**Background:**

Physicians’ communication skills are important for patient-centered care. Although working with simulated patients (SPs) in case simulations is common for training communication skills, studies seldom include a wide range of challenging behaviors or explore students’ own experiences of learning communication skills with SPs. Therefore, this study was aimed at investigating how medical students perceive communication training involving challenging consultations with SPs and the impact on their learning experiences.

**Methods:**

Twenty-three medical students from the same class were interviewed in focus groups about their experiences of simulation training with actors as SPs. In the simulation training, the students were instructed to deliver bad news, manage negative patient reactions, and encourage behavioral changes in reluctant patients. This was followed by feedback and a debriefing exercise. The interviews were analyzed with content analysis.

**Results:**

Students reported that actors as SPs made the simulations more realistic and enabled them to practice various communication skills for challenging consultations in a safe way and manage their own feelings, thereby promoting new learning experiences. Elements such as actors’ flexibility in changing behaviors during role-play and exposure to different challenging behaviors, like negative emotions, were regarded as valuable. The importance of an accepting and permissive climate for the debriefing exercise was highlighted, though without taking too much time from the simulation training. Feedback directly from the SP was appreciated.

**Conclusions:**

Actors as SPs were perceived as a valuable part of challenging communication training and added elements to the learning process. Future studies should include a wider range of challenging behaviors in training with SPs and evaluate the effects of such training on students’ use of communication skills.

**Supplementary Information:**

The online version contains supplementary material available at 10.1186/s41077-022-00230-3.

## Background

Communication skills are regarded as an important element of the medical consultation and make up part of patient-centered care [1–4]. Communication skills encompass several competencies. Among them is active listening techniques, such as encourage the patient to start and continue to talk, the use of open-ended questions, to use repetition/reflection, and summaries of both what the patient said and of emotions expressed or shown by the patient [2, 4]. Communication skills also include aspects of empathy, i.e., understanding patient experiences, concerns, and perspectives, combined with a capacity to communicate this understanding [2, 5, 6], and motivational interviewing (MI), a patient-centered method developed to promote behavioral changes in a patient, with a focus on exploring patient ambivalence [7].

Some consultations are more challenging than others, including presenting bad news to a patient or talking to an angry or unmotivated patient. If information is delivered in an inadequate way, it may affect patient outcomes or result in increased suffering, misunderstanding, or bitterness in the patient, whereas if it is delivered well, this can help the patient understand, accept, and adjust to the information [8]. Dispiritingly, some studies have reported that medical students’ empathy decreases during medical school [5, 9]. Specific aspects linked to a decline in empathy levels include feelings of pressure or exhaustion and the need for detachment from the more emotionally challenging aspects of medicine [10, 11]. Previous research has shown that delivering bad news to patients provokes more cardiovascular and perceived stress among medical students [12], and students may instinctively use fewer communication skills in such situations. It is essential for medical students to recognize patient anger or displeasure and to learn techniques for de-escalating such situations [13].

Simulated patients (SPs, in the literature, the term standardized patient is sometimes used) have been recommended for training communication skills in case simulations [14] and are now often involved in education at medical schools [15–17]. SPs are usually actors who have been trained to portray patients and are engaged by medical educators in both formative and summative objective structured clinical examinations [17]. By working with SPs in education, healthcare providers are given the chance to improve their patient relationship competencies, such as using active listening techniques and empathic responses, in a safe and realistic way. Accordingly, medical students have been found to express increased comfort with addressing difficult topics with patients after having met SPs in their education [18, 19]. Previous research has also found that working with SPs in communication skills training among undergraduate medical students can improve both nonverbal behaviors, such as open body positions and adequate facial expressions [20], and verbal behaviors, including use of open and closed questions, encouragement of patient responses and emotions, and invitations for clarification [21].

There is growing literature focused on involving SPs in the training of communication skills, where most studies focused on a selected group of skills, e.g., taking a clinical history [22], delivering bad news [18, 23], managing strong emotions [24], or had SPs as examiners of students’ communication skills [25]. Studies seldom explore medical students’ own experiences of learning communication skills with SPs, which may be of great importance when exposed to challenging behaviors, where communication skills are put to the test. In a review on simulation-based methodologies used among undergraduate psychiatry medical students, it was concluded that few studies have explored the impact of learning experiences with SPs, with students being asked to reflect on their own performances in simulations or how simulations facilitated their learning [26]. In the present study, we implemented a teaching method for training patient-centered communication skills in a wide range of challenging situations, working with SPs during the eighth term in the medicine program. The study aim was to investigate how medical students perceive challenging communication training with SPs by interviewing students about their experiences of the training and learning.

## Methods

### Procedure and participants

All students at the medicine program at the Uppsala University receive training in communication skills during the first, fourth, and eighth term. The medicine program at the Uppsala University extends over 11 terms (5.5 years), of which terms 1–4 are regarded as preclinical and 5–11 as clinical. During the eighth term, in which the students learn about psychiatry, child and adolescent psychiatry, neurology, ophthalmology, and otorhinolaryngology, a 3-day course, *Communication skills in challenging situations*, is held. When the students start this term, they master active listening techniques as a way to start a consultation, but not how to use them in challenging situations. The 3-day course comprises lectures on communication skills (3 h), including theory on conversation skills needed for patient-centered communication in challenging situations, such as how to use active listening and empathy in challenging situations and how to motivate a patient to change a behavior by using techniques from MI. Following the lectures, students first discuss a recorded role-play in small groups including peers and a supervisor (3 h). Then, the students role-play with actors as SPs in small peer groups (4 × 4 h), to practice the necessary skills. All students from one class were invited to focus groups after the course, at the end of the term, to explore their experiences of working with SPs. In total, 23 (27%, 13 females) agreed to participate (split into three focus groups: *n* = 9, *n* = 11, and *n* = 3). The interviews in the focus groups were conducted on different days. The students could choose when they wanted to participate a few days in advance, meaning that the numbers of students in the different focus groups varied. The study was approved by the Swedish Ethical Review Authority, Dnr 2019-05908. All subjects involved provided informed consent for participation and publication.

### Training with simulated patients

The simulations were conducted in groups of six to eight students, one SP (a professional actor who has been trained to portray patients consistently) and one supervisor for each scenario. The student groups moved between four rooms, each of which presented a different scenario. The student groups spent 90 min in each room, and two to four students got the chance to act as the doctor for each specific scenario. All students role-played as a doctor in one or two scenarios. The four scenarios were as follows:

1. Telling an elderly woman with aggressive colon cancer that she is dying, using empathic responses
2. Managing negative patient reactions, such as anger and anxiety, with a woman who is unhappy with the care she has received and who suspects that she has been discriminated and trying to find a way forward, using active listening
3. Discussing a rehabilitation plan with a reluctant middle-aged man with back pain who has been on paid sick leave and wants his sick leave certificate prolonged, using MI tools and active listening
4. Motivating a reluctant young man with alcohol problems to change his lifestyle, using MI tools and active listening.

All scenarios were complicated by negative patient reactions. For a more detailed description of the scenarios, see Supplementary Table 1. As a post-simulation debriefing exercise, the student acting as the doctor reflected on his or her perceptions of the role-play, as did the other students. The student acting as the doctor also received immediate verbal feedback after the role-play from the supervisor, as well as from peers and the SP.

The student and the supervisor could ask for a time-out during the role-play if needed. During the post-simulation debriefing and feedback session, all those who participated were encouraged to discuss the following questions: “What did they feel during the consultation?”, “What worked well in the consultation?”, “What could have been done differently in the consultation?”, and “What had they learned?” Each role-play lasted for approximately 15 min and the post-simulation debriefing for approximately 15 min. To ensure the psychological safety of the learners and the SPs, we included the option of a time-out; we had a template for both debriefing and feedback, where positive feedback was given first; and the supervisor could meet with each student afterwards, if deemed necessary. All supervisors were experienced medical doctors and psychologists trained in supervision.

### Focus groups

The interview guide included the following questions: “How did you experience participating in the simulation training?”, “What do you perceive that you were practicing?”, “How did the simulation training change your communication skills?”, and “How did the simulation training affect your patient relationships?” Follow-up questions were asked when appropriate. The interviews were audio-recorded and lasted for about 32–48 min and were then transcribed verbatim and pseudonymized (though sex was retained). The interviews were performed by one graduate medical student (JK), who interviewed students in two focus groups, and one psychologist, who interviewed one group. Both interviewers were females. The participants did not get any specific information about the interviewers. The researchers (JI and MR) were involved in the education and informed the students about the research project. The interviewers did not participate in the education/simulation training, and since they had no training in interviewing focus groups, they were trained by one of the authors (JI).

### Data analyses

Based on the text from the focus group interviews, a qualitative content analysis was carried out using an inductive approach, where categories were derived from the data [27]. The analysis was conducted by two of the authors (JI and MR), who continuously discussed and confirmed the findings. First, the material was read to achieve an optimal understanding of the content. Second, all meaning units, defined as one or more sentences or just part of a sentence carrying a meaning connected to the research question, were extracted. Third, the meaning units were shortened to their essence. Fourth, text units with similar meaning were grouped into mutually exclusive categories. Fifth, categories were divided into subcategories based on dissimilarities within the categories. To increase the rigor of the analysis, the interview text was reread, and the categories and subcategories were compared and validated against the text. No software for qualitative analysis was used to process the data.

## Results

As shown in Table 1, the qualitative analysis resulted in seven categories and 20 subcategories. These are presented below with quotes that reflect the subcategories.

**Table 1:** Categories and subcategories in the qualitative analyses

Categories	Subcategories
Debriefing and feedback	Receiving feedback
	Basis for good debriefing and feedback
	How much time spent on debriefing and feedback
	During or after simulation
	From whom?
Working with SPs	Realistic
	Unrealistic
The cases	Implementation
	Flexibility of the actors
The learning process	To practice challenging consultations
	Amount of training
	To manage own feelings
Lessons learned	Specific techniques
	Increased experience
	Changes in communication skills
Relevance	Relevant
	Irrelevant
Suggestions for change	Offered earlier
	Smaller groups
	Other cases

### Debriefing and feedback

The subcategory *Receiving feedback* included statements about feedback received within the groups, where concrete suggestions on how to change behavior were regarded as very helpful. In *Basis for good debriefing and feedback*, the students discussed the importance of having a common understanding of, and format for, how to reflect on experiences and how feedback should be delivered and the need for an accepting and permissive climate in the group. As one student commented, “the strength of the course is that you expose all your limitations in communication skills in front of ten people, and that obviously requires a supportive learning climate” (man, group 2). In *How much time spent on debriefing and feedback*, some thought it was good with a lot of feedback, while others thought too much time was spent on this part, especially when all the students were asked to give feedback and express how they felt, and the same comments were repeated. One student said that at one station, “two students were doctors and then we discussed how they had behaved for like 45 minutes, it was awful, whereas at another station we were given the chance to practice and then more students could practice” (woman, group 2). Comments were made on whether feedback should be given *During or after simulation*, with some students describing the benefits of getting feedback during the simulation, so changes in relation to the SP could be implemented immediately: “we could pause the situation, and then kind of … I would kind of change my body position. And then she reacted to it … it was perfect” (woman, group 3). Others highlighted the risk of interruptions interfering with the consultation progress. The last subcategory, *From whom?*, showed that feedback from the SP was highly appreciated.

### Working with SPs

It was discussed that working with SPs (actors) made the simulation more *Realistic* as compared with role-playing with fellow students. One student said: “It felt immediately like meeting a real patient. That is, I was thinking it was a patient, and I got that feeling as well. It was an excellent practice” (man, group 2). Another student said that the training “adds another dimension. It is as close as you can get to meeting a real patient. It is someone you don’t know and don’t have a relationship with” (woman, group 1). Some thought that the simulation training was constraining or that the SP became too extreme, and that it was *Unrealistic* and did not feel like having a real patient.

### The cases

In the subcategory *Implementation*, the students expressed appreciation for the opportunity to choose what emotions, e.g., angry, sad, and manipulative, the SP should portray. There were comments about how the supervisors led the sessions, and the students reacted negatively when the supervisor was not prepared or talked too much. The opportunity to ask for a time-out and get help from the group and the supervisor was appreciated. However, students were more critical when the supervisor asked for a time-out since it disrupted the simulation training. In *Flexibility of the actors*, the students emphasized the benefits of the SPs being able to change the portrayed character, so that the next student acting as the doctor did not have to repeat the exact same simulation.

### The learning process

The students discussed that the simulation training with actors enabled them *To practice challenging consultations* in a safe way. Some took the opportunity to practice managing emotions expressed by patients that they felt were hard to deal with, e.g., anger. One student said: “I felt it was ... special to see a patient who became really angry, first angry and then furious. I am not used to dealing with people’s anger, so it is very good to be able to practice” (woman, group 2). Being exposed to strong emotions was mentioned as an educational experience, and one student said that it was sometimes hard to remain calm and professional. Some students mentioned that it was useful to experience not being able to effectively manage the SP’s reactions, for instance not being able to provide reflections and summaries because of the anger expressed by the SP, and not knowing how to react to the SP’s reactions. The experience of delivering bad news to the SPs was also appreciated by the students. In the subcategory *Amount of training*, the opportunity to test or observe different challenging consultations was described as positive. Some argued that it would have been good to act as the doctor in more consultations. Another learning process discussed was *To manage own feelings* in the consultations: “To manage one’s feelings, to deliver bad news, read the body language, to use one’s own body language. To sit closer to someone who is sad, keep a respectful distance to someone who is angry …” (woman, group 2).

### Lessons learned

*Specific techniques* that the students discussed as being practiced in the simulations were motivational efforts (working with the SP’s ambivalence) and delivering bad news. Another consequence of the simulations was *Increased experience* and the students described being better prepared for the future, thanks to having practiced challenging conversations: “The day when I meet a patient who is very angry with me, I will be happy that I have at least met an actor who was really angry with me” (woman, group 1). It was stated that simply having established a contact with the patient was acceptable, and that you could not always reach a solution: “Then I will do my best … and even if it is not completely under my control, that’s okay” (man, group 1). *Changes in communication skills* that were discussed were the experience of having better conversations after training, a better ability to create an alliance with a patient and to keep calm, and being better at giving feedback to peers.

### Relevance

It was discussed if the simulations with SPs were *Relevant*; especially, those who had worked with psychiatric patients felt that they were. Others stated that some skills were *Irrelevant*, such as delivering bad news which might not be relevant for many years. Others felt that these skills could not be fully practiced in the clinic due to lack of time and the inappropriateness of a student doing it, and some thought that the extremely challenging consultations practiced during this course were not common in real life. One student was concerned that using active listening could make the medical doctor become too permissive and feel unable to say no to a patient.

### Suggestions for change

Some suggested that the simulations with SPs should have been *Offered earlier*, before the course in psychiatry or even earlier in the program. One student asked, “why did we not have this earlier, why do we have it now, four years into the program?” (woman, group 3). The need for *Smaller groups* was discussed, with more time for each student to practice. In order to minimize discussions on who should role-play the doctor in a simulation, it was suggested that this could be decided beforehand. Talking about suicide and giving bad news to a relative were suggested as *Other cases*.

## Discussion

The use of simulation methodologies with SPs in medical education has increased greatly in recent decades and has been suggested to be a key determinant of a student’s success in learning, with safe, active, and collaborative learning environments regarded as crucial [26]. In this study, we explored how medical undergraduate students perceived working with actors as SPs in simulation training for various challenging situations. The qualitative analysis from the focus groups showed that feedback from SPs was appreciated, and that working with actors generally made simulations more realistic, as compared with peer role-play. At the same time, others emphasized the need for an accepting and permissive climate when working in groups and said that too much time was spent on feedback and debriefing, which interfered with the simulation training. Elements such as actor flexibility in changing behaviors and being exposed to different challenging consultations and emotions were regarded as valuable and educational. Students described feeling more prepared for having challenging conversations in the future.

Previous studies have seldom included training of several communication skill techniques for a wider range of challenging situations such as dealing with angry, anxious, or unmotivated patients, delivering bad news, and using negotiation tactics. This is of great interest, since communication skills are put to the test in such instances. In the interviews, the students stated that the training with SPs enabled them to practice challenging consultations in a safer and more patient-centered way and to manage their own feelings when exposed to negative patient reactions, giving them new learning experiences. These reflections correspond well to models of adult learning where focus should be on creating a safe and active environment and where the transformative learning process should include emotional elements, encourage critical self-examination of the situation causing the discomfort, and development of new ways of thinking about and managing the situation [26, 28]. Corroborating this, some students mentioned that feedback and discussions during the simulation sometimes resulted in immediate changes within the consultations, while some described having better conversations and a better ability to create alliances with patients after the training.

Feedback, especially from the SPs, was mentioned as an important element, but also as something that created stress, especially if the group climate was not constructive. A trusting and collaborative climate has been emphasized as an important prerequisite for learning [28]. Some said that too much time was spent on debriefing or feedback and wished for more practice with the SPs. In line with this, students suggested smaller groups with more time for each student to practice. Simulations with SPs were generally regarded as relevant, and it was suggested that this should have been offered earlier in the medical program. One concern raised was that the communication skills practiced during the course might not be applicable later in a real-life setting, when working as a medical doctor, since there would not be enough time allocated with patients.

Previous research has reported that students express appreciation of working with SPs in teaching and learning [29] and state that having encounters with SPs increases their comfort in addressing difficult topics with patients [18, 19]. Working with SPs in simulations has been reported to be more effective in increasing self-confidence among nursing students than peer role-play [30]. Previous research has discussed the limitations of peer role-playing, which depends on students’ abilities to portray patients and give realistic responses [14, 30]. In our study, the flexibility of the actors in changing character was highlighted as important for the learning process. Furthermore, the students expressed appreciation for the actors’ flexibility and getting the opportunity to choose what emotions the SP should show, e.g., anger. Research on how SPs are perceived by students has shown mixed results: interprofessional healthcare professionals and teachers stated that exercises with SPs were associated with an increased sense of realism, and actor feedback was highlighted as a key component in learning [31]. Others have reported that medical students found interactions with SPs to be unnatural [32] or felt that actors overacted and did not behave like real patients [33], although this view was not shared by teachers [33]. In our focus groups, working with actors as SPs was generally regarded as increasing the sense of realism in the simulations, although a few experienced it as constraining and stated that the actors were too extreme and overacted.

This study has some limitations. First, since this is an exploratory study, no conclusions about the effects of working with SPs in communication training can be drawn. Rather, the aim of the study was to explore how students experienced the training, for which interviews and qualitative content analysis were seen as appropriate methods [34]. Second, the researchers who performed the analysis also served as supervisors in the training, and previous knowledge of the participants could potentially influence the interpretation of the text. However, pseudonymization of the transcribed text decreased the risk of linking the text to specific persons. Furthermore, those who conducted the interviews did not participate in the education/simulation training. Of the two interviewers, one was included as an author in this article and was writing a student essay on data collected from the medical students, which may have increased the risk of confirmation bias. However, the essay was based on quantitative data collected from the medical students in a separate project; hence, the interview data were not included in her project. The other interviewer had no other association with the project. The researchers were involved in the education and also invited the students to the focus groups, which could have resulted in some selection bias, as only those positive to the course may have volunteered to participate. In order to limit this bias, the invitation was issued, and the interviews conducted after the course had ended, and the students were informed that no personal data would be saved, that data would be pseudonymized, and that participation was voluntary. Third, data saturation was not used in the recruitment procedure. However, the number of participants (*n* = 23 in three focus groups) is rather large for a qualitative study, and we found that the last interview confirmed previous results. Fourth, there was a high attrition rate, which may have resulted in selection bias. We also lack data on specific characteristics of the sample, such as age and prior work in the healthcare setting. Fifth, since we used transcript data at a group level, it was not possible to identify participant quotes with participant numbers. However, it was clear from the transcripts that a significant number of included students — and all focus groups — contributed to the data. The findings should be regarded as preliminary, and further research is warranted to confirm the categories and recommendations.

## Conclusions

We conclude that including SPs in communication skills education among medical students was appreciated and seemed to add important aspects to the learning experience, including an increased sense of realism. When working with SPs in education, a flexibility of the actors to change character, offering the students a choice of negative emotions, and giving them the opportunity to practice different types of challenging situations seem to be of importance. Otherwise, the simulations may become too repetitive. The debriefing and feedback exercise should promote discussions within the group and constructive feedback, with SPs being encouraged to participate. Still, the debriefing and feedback should be focused and short, not taking too much time from the simulation training, as the opportunity to test and observe a number of challenging consultations was experienced as particularly positive. As it is essential to train future medical doctors in communication skills, evaluation and examination of these skills and how they are best taught are of utmost importance. The effects that simulation training involving challenging conversations with SPs have on the use of communication skills need to be further explored. Using randomized control designs and/or longitudinal designs would be preferable, to evaluate the effects of simulation training on communication skills and patient outcomes over time.

## Supplementary Information

**Additional file 1: Supplementary Table 1.** Summaries of the standardized patient cases, with main goal and suggested use of communication tools.

## Data Availability

The data that support the findings of this study are available on reasonable request from the corresponding author. The data are not publicly available due to privacy or ethical restrictions.

## Abbreviations

MI: Motivational interviewing
SP: Simulated patient
