# Supplementary material for: Medical students’ experiences of working with simulated patients in challenging communication training
Source: Adv Simul (Lond). 2022 Oct 10;7:32. doi: 10.1186/s41077-022-00230-3 (PMC9552443; doi:10.1186/s41077-022-00230-3)
Supplement: Supplementary file 1 — Additional file 1: Supplementary Table 1. Summaries of the standardized patient cases, with main goal and suggested use of communication tools. [file 41077_2022_230_MOESM1_ESM.docx]

**Supplementary Table 1** Summaries of the standardized patient cases, with main goal and suggested use of communication tools

| Case 1. An elderly woman who has aggressive colon cancer, which is being treated palliatively with cytostatics. The latest ultrasound showed that the cancer has spread to the liver and that the liver enzymes are rising. The doctor has concluded that the patient is dying and is in a critical state. In this case, the main goal is to deliver the news to the patient that she soon will die and to express empathy for the patients’ situation. |
| --- |
| Case 2. An elderly woman who is unhappy with the care she has received from her doctor. She suspects that she has been discriminated because of age and sex and worries about having a heart problem, despite that she has been thoroughly examined and all tests have come back normal. She has now booked a consultation with a senior doctor, but because of a rebooking she instead meets a young doctor. The doctor suspects that the heart problems the patient is experiencing are panic attacks. In this case, the main goal is to handle negative patient reactions and to try to find a way forward, mainly by the use of active listening. |
| Case 3. A middle age man with back pain, who has been on paid sick leave on and off for the last two years. The patient is sceptical to physical therapy and wants his sick leave certificate prolonged. The doctor considers the patients’ best chance to rehabilitation to be through physical therapy and does not see a reason for the patient not to be able to work. In this case, the main goal is to discuss with the patient about sick leave, by using MI-tools and finding common ground for a rehabilitation plan. |
| Case 4. A young man who has contacted his local health care centre because of stomach pains and fatigue. During the first doctor’s consultation it becomes clear that the patient has a high consumption of alcohol, a high blood pressure and elevated liver enzymes. The patient claims that he has a normal alcohol consumption and denies any comments about high consumption. During the simulation the student will inform the patient about the test results from the earlier visit. In this case, the main goal is to motivate the patient to change his lifestyle, by using MI-tools. |
